# Supplying whole blood with drones for prehospital transfusion at trauma sites in Finland: A simulation

**DOI:** 10.1111/vox.70092

**Published:** 2025-08-13

**Authors:** Panu Erästö, Milla Juntunen, Jukka Pappinen, Jouni Nurmi, Jarkko Ihalainen, Jouni Lauronen, Mikko Arvas

**Affiliations:** ^1^ Department of Information and Service Management Aalto University School of Business Espoo Uusimaa Finland; ^2^ Finnish Red Cross Blood Service Vantaa Finland; ^3^ Faculty of Medicine, Department of Public Health University of Helsinki Helsinki Finland; ^4^ Emergency Medicine and Services Helsinki University Hospital and University of Helsinki Helsinki Finland

**Keywords:** blood product, drone, optimization, prehospital transfusion, simulation, trauma, whole blood

## Abstract

**Background and Objectives:**

Prehospital transfusion is now increasingly used in civilian and military medicine. Blood products are, however, perishable and rarely needed in civilian settings. Given the rapid development of drone‐based logistics and Finland's low population density, we aimed to build a computational framework to assess the feasibility and requirements of drone‐based delivery of blood products to trauma sites. Unlike previous studies, which focus mostly on deliveries to hospitals in compact urban areas, we model direct deliveries to trauma scenes across an entire sparsely populated country.

**Materials and Methods:**

We used predicted trauma data on a 1 × 1 km grid covering Finland. Drone base locations were optimized using mixed‐integer linear programming, and drone operations were analysed with a discrete event simulation model. Our approach combines strategic location optimization with operational‐level simulation and is grounded in high‐resolution, real‐world data‐driven trauma demand estimates.

**Results:**

With 20 base locations and drones capable of a 60‐km delivery range, over 80% of predicted trauma events can be reached. If drones can return to base, one drone per base is typically sufficient due to the rarity of missions.

**Conclusions:**

We present a novel computational framework for simulating drone‐based blood delivery to trauma scenes. Our results suggest that while current drone capabilities may still be limited, the approach is promising for countries with similar geography. The framework is adaptable and can support planning in other regions with reliable trauma demand data.

**Highlights**

- We present a novel computational framework that combines strategic location optimization and operational‐level simulation to model drone‐based blood delivery directly to trauma scenes across a sparsely populated country.
- Using real‐world trauma demand estimates on a 1 × 1‐km grid covering Finland, we show that 20 drone‐equipped blood distribution centres with a 60‐km range can reach over 80% of predicted trauma events.
- Due to the low frequency of trauma events, a single drone per distribution centre is typically sufficient, making the system operationally efficient even in sparsely populated regions.

## INTRODUCTION

Prehospital transfusion has become increasingly common in civilian and military medicine [1, 2]. The clinical benefit of early transfusion with whole blood has been shown for military casualties [2], while the results are ambiguous in the civilian sector [3]. When considering the cost–benefit ratio of prehospital transfusion, the logistics of products and time for treatment are significant factors [4]. Since blood products are perishable, storage and distribution need to be carefully planned to avoid wastage while reaching clinically optimal availability of blood [5]. In Nordic countries, the demand for whole blood within the emergency medical community has been increasing. Accordingly, the Finnish Prehospital Whole Blood study (FinnPHWB, NCT NCT05744583) studies the clinical benefit of prehospital transfusion with leukoreduced, low‐titre, group O RhD positive, male donor whole blood (LTOWB) in Finland in conjunction with this study.

During the last 10 years, drones have been increasingly utilized in various emergency care applications, including the transportation of blood products [6, 7]. The use of drones for transporting medical supplies, including blood products, to hospitals has been studied and even implemented in some regions, both at broader and more localized levels [8, 9, 10]. However, the direct delivery of blood products from blood distribution centres (BDCs) to field locations—such as accident scenes or rural prehospital settings—using drones remains largely unexplored. To date, no published studies have assessed the feasibility or clinical impact of such prehospital deliveries on a wider scale in the civilian sector, leaving a significant gap in the evidence base for this potentially transformative approach.

In the context of prehospital blood transportation, the planning of optimal locations for BDCs and the number of drones with respect to performance measures by drones are key factors in the performance of such applications. Relatively sparsely populated regions, with population density for example below 50 inhabitants per square kilometre, cover at least half of the global surface. In these regions, unique logistical constraints exist, and the need for optimization of BDC locations is particularly pronounced. This research aims to model the combination of delivery and demand of prehospital transfusion with drone logistics to support the future development of the Finnish blood supply chain. With 19 inhabitants per square kilometre, Finland provides a good example of a sparsely populated country. This research also provides an example and the required computational tools to carry out similar analyses in other countries. Optimization methods and simulation have complementary strengths in the analysis of supply chains [11, 12], and we have used both approaches.

We acknowledge that several practical factors—beyond the locations of BDCs and drone specifications—may influence the feasibility and effectiveness of drone‐based blood delivery. These include geography, air traffic conditions, legal and regulatory requirements, climate, blood unit discard rates and the risk of drone signal interference. For further discussion of these factors; see for example References [13, 14]. While these considerations are essential for real‐world implementation and operational planning, the primary focus of this study is on strategic modelling of the performance of prehospital transfusion scenarios at the scale of a nationwide system.

## MATERIALS AND METHODS

### Data

As data we used count of predicted traumas for both 1 × 1 km and 5 × 5 km non‐overlapping grid maps of Finland [15]. As trauma we considered accidents that required medical attendance from an emergency physician while transporting the patient to a hospital, which could also indicate a potential need for prehospital blood transfer. The trauma predictions were calculated based on a previously conducted Finnish government‐funded study on the current state and cost‐effectiveness of Helicopter Emergency Medical Service (HEMS) in Finland [16]. In brief, the prediction model is based on the incidence of severe trauma missions in the vicinity of HEMS bases from Finnish National HEMS (FinnHEMS) mission database, the geographical distribution of trauma missions dispatched by Emergency Response Centres (ERCs) and historical data of road traffic accidents in each region by road type.

### Optimization of BDC locations

To optimize the locations of BDCs to act as bases for drone operations to reach as many trauma scenes as possible, with given parameters, we used the tool given in Reference [17]. This computational tool determines the optimal locations for BDCs in order to fulfil blood product orders from nearby hospitals by applying a mixed‐integer linear programming approach, and it is a robust choice due to its open‐source and free accessibility as well as its proven track record in previous studies. In our setting, the ordering hospitals in the original setting were replaced with the 5 × 5 km squares (which are later called trauma scene locations) and count of predicted traumas over 10 years due to computational requirements.

As BDCs we used 25 Finnish major hospitals (Tables S1–S5). The direct distance from each hospital to the centre of each square kilometre was calculated by QGIS 3.28 software along a great circle.

To run the ‘facility_location_bloodsupply’ tool of Reference [17], a script was coded to create required input files for optimization. The script creates a file (Dt.csv) containing flight times between all of the hospitals and trauma scene locations. These were calculated assuming that the drone's average flight speed (net speed) is 100 km per hour. This represents total flight time, that is, including take‐off and landing. The other input file (F.csv) contains locations of the hospitals and trauma scenes. Instead of the delivered products used in the original setting, the predicted traumas were used to create the required ‘pickle’ files. Subsequently, the optimization was performed with settings OBJ = ‘time’, transports = ‘direct’, source = ‘E’ and ORDER_DATA = True, *N* = (5, 10, 15, 20, 25), P_CAT = FALSE, ROD_TIME = 30 min. The parameter values were chosen to simulate transportation of blood products to trauma scenes with drones.

### Simulation of drone blood delivery

Given sets of BDCs to act as bases for drone operations, we developed a simulation model to generate trauma events on a more fine‐grained 1 × 1 km non‐overlapping grid map of Finland. For each 1 × 1 km square, predicted intensities of one‐year periods were used as trauma probabilities. This model replicates real‐world conditions in which demand for blood product distribution arises in specific geographic regions. The inter‐arrival times of these events were modelled using an exponential distribution, reflecting the random temporal distribution of delivery needs. To account for variability and reduce the impact of randomness from any single year, the simulation was executed independently 1000 times and the results given later were calculated as averages over these simulations.

In the simulation, events were managed using the following approach: each event within a drone's range was handled to ensure the fastest possible response. This involved optimizing the drone's route and schedule to minimize the arrival time at the event location. If the nearest drone was occupied at the time of an event, the system selected a quicker solution from two options: (i) queuing at the nearest BDC until the completion of the drone's current mission or (ii) dispatching an alternative available drone closest to the target location within range. Drones were assumed to be occupied only for the round‐trip travel time to the event location, with no delay time considered at the destination, for simplicity. Travel time depended on the distance to the destination and the drone's speed, which was assumed to be constant throughout the route.

## RESULTS

### Optimization of blood delivery centre locations

**FIGURE 1:**
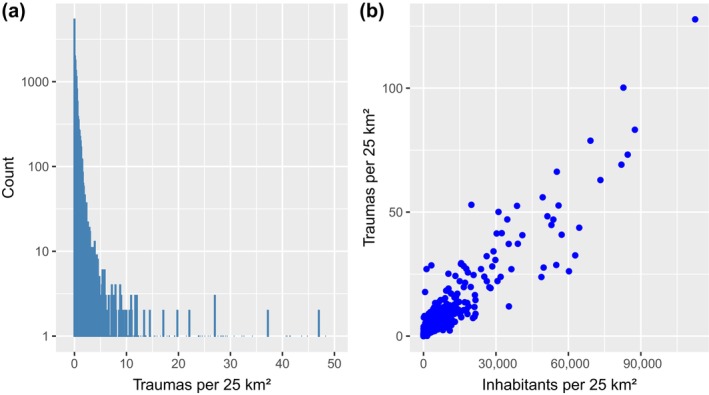
(a) Distribution of predicted traumas per 10 years for 5 × 5 km squares, that is, accidents that required medical attendance from an emergency physician while transporting to hospital. The *x*‐axes has been truncated at 50 to enable visualization. There were 13 5 × 5 km squares with above 50 predicted events per 10 years. (b) Correlation of count of predicted traumas (a) and inhabitants per 5 × 5 km squares.

**FIGURE 2:**
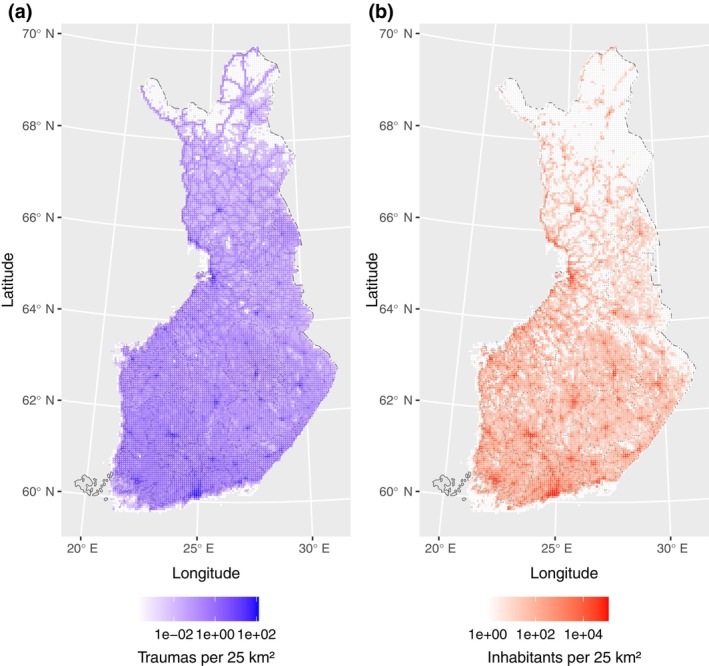
Population density of Finland (a) and the distribution of predicted traumas per 10 years (b) on 5 × 5 km squares in Finland.

A total of 13,245 trauma scene locations with the number of predicted traumas greater than zero were included in the optimization (Figure 1). The predicted count of traumas per 10 years in a 5 × 5 km square shows a heavily right‐tailed distribution with a median of 0.18 (1st quartile 0.018, 3rd quartile 0.48 and 2381 squares with zero counts) (Figure 1a). The predictions are mainly driven by population density (Figures 1b and 2). The count of inhabitants and the count of predicted traumas have a correlation *r* = 0.94. In addition to population density, major roads, that is, intensity of traffic, contribute notably to trauma count predictions (data not shown) (Figure 2).

**FIGURE 3:**
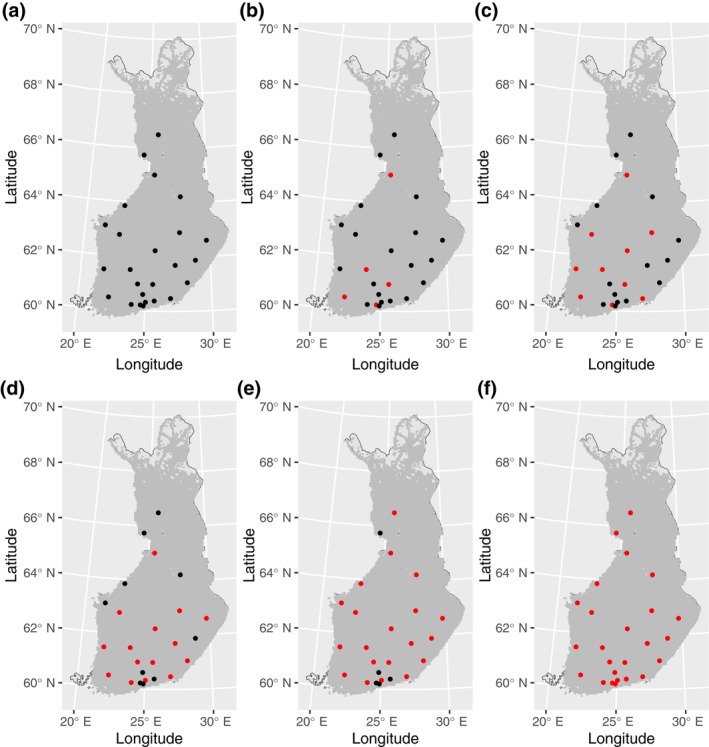
Optimized selection of blood distribution centres (BDCs) with different total number of BDCs and with a flight time of 30 min on the map of Finland. Set of candidate BDCs are shown in black and those selected by optimization in red. Used BDC set sizes was 5 (b), 10 (c), 15 (d), 20 (e), 25 (f).

In Figure 3, the optimized selection of BDCs with different numbers of BDCs (*N*) and with a one‐way flight time limit of 30 min is plotted on the map of Finland (for full details see Table S1). The selected BDCs are in line with the predicted trauma intensities and population density (Figure 2). With a small number of BDCs, the locations are concentrated in the south and west of Finland while increasing the number of centres makes more sparser areas from eastern and northern Finland be included. These are in line with the results in Reference [5].

### Simulation of drone blood delivery

In the following, we report the results from the simulation model using the optimized locations with different number of BDCs and by considering the different values of drone parameters (speed and half range). We consider a range of parameter values reflecting current and near future capabilities of drones. For simplicity, we assume that the correct blood product is always available, the drone is dispatched from a BDC without any delay, flies directly to the target area at a constant speed and is able to land instantly. The results are presented from two perspectives: first, from the patient perspective, focusing on the service's reach and response times; second, from the managerial perspective, emphasizing the technical and operational specifics of the implementation.

**FIGURE 4:**
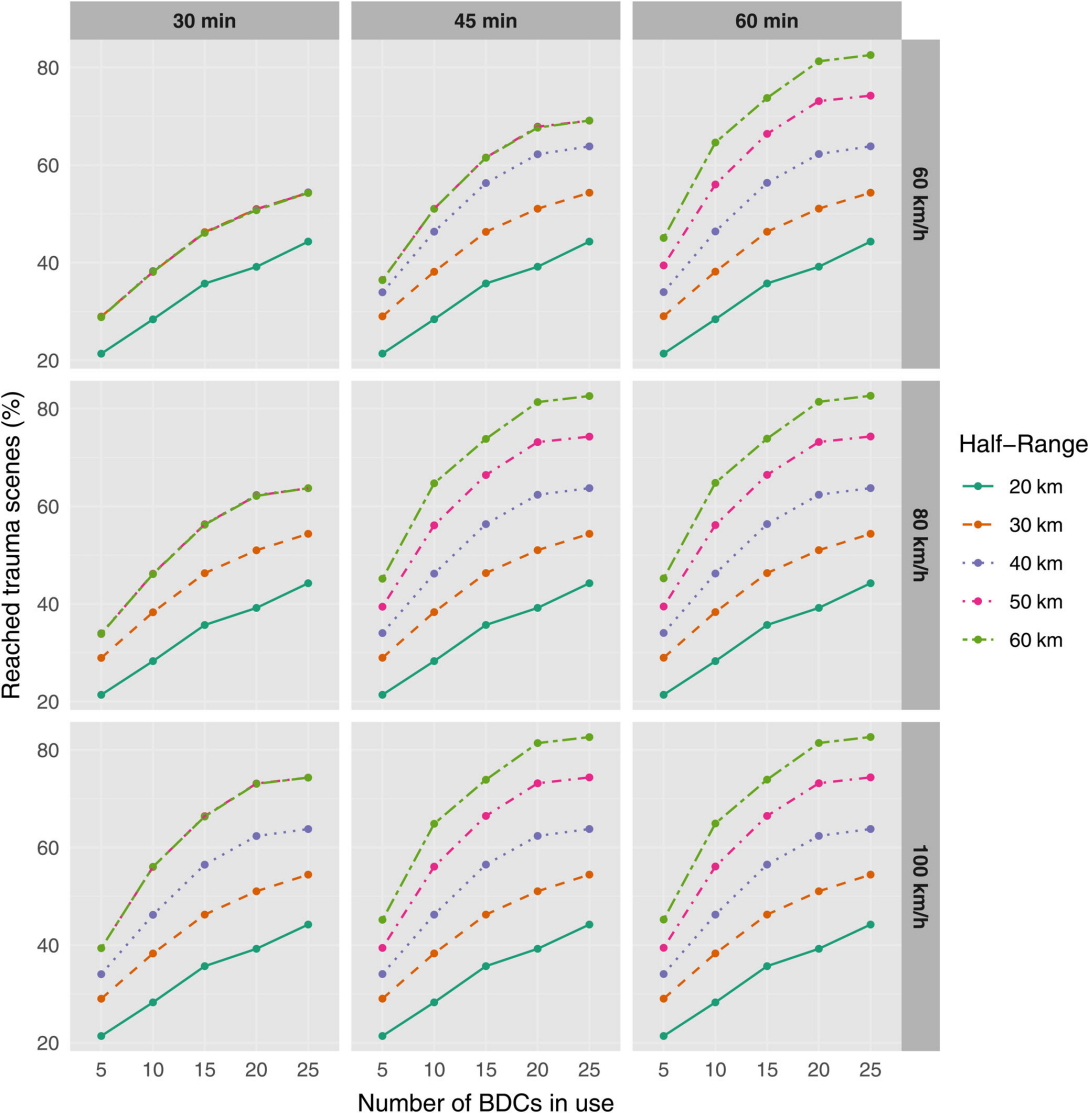
Percentage of trauma scenes reached as a function of the number of blood distribution centres (BDCs) in use, time limit (columns), drone speed (rows) and drone half range (colours).

**FIGURE 5:**
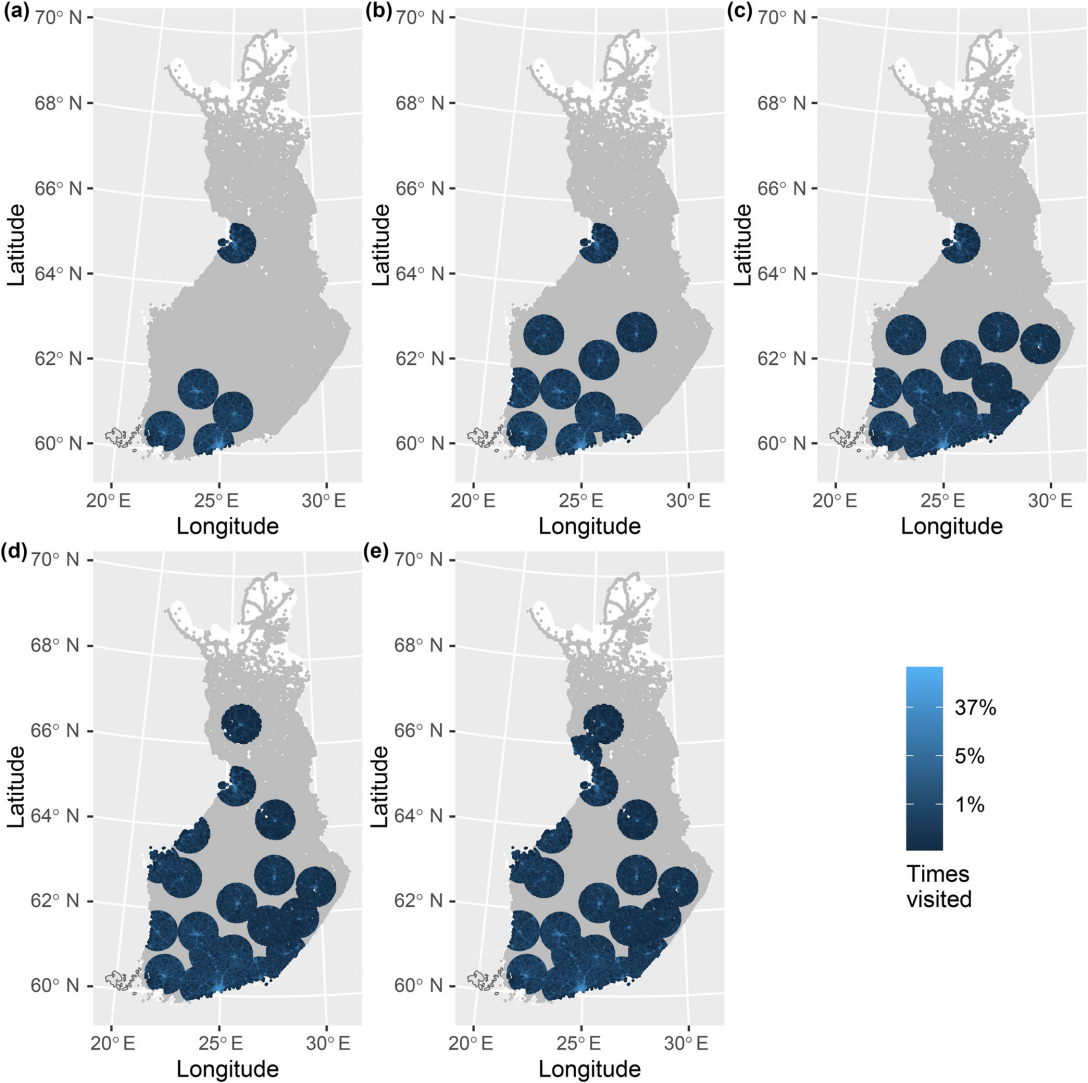
Illustration of a single line from Figure 4, that is, speed 100 km/h, half range 50 km and maximum time 30 min on map of Finland. Used blood distribution centre (BDC) set sizes was 5 (a),10 (b),15 (c), 20 (d), 25 (e). In each panel ~30% of 1 × 1 km squares are visited only once by a drone in all the 1000 simulations, which corresponds to the darkest blue colour and percentage 0.01. Grey colour corresponds to trauma scenes never visited and white colour to squares with no traumas.

From a clinical perspective, the delivery times of the service are of utmost importance. However, it is difficult to determine a single choice of time limit for completing the deliveries. Therefore, we report the main results using three different time spans. Figure 4 shows the proportion of trauma scenes reached within different one‐way flight time limits (30, 45 and 60 min) and with varying numbers of BDCs, along with different drone parameters for speed and half range. Figure 5 overlays a single parameter set from Figure 4 with varying BDCs on a map of Finland. With the shortest time limit (30 min), the proportion of reach varies between 21.4% and 74.3%. In contrast, with the longest time limit (60 min), the proportion varies between 21.4% and 82.6%, depending on the number of BDCs and drone parameters.

As expected, the percentage of trauma scenes reached generally increases as the selected time limit increases or any of the three explanatory variables increases. The number of BDCs typically improves reach, except in some combinations with high values of BDCs and a half range of 60 km, where the increase is only marginal with time limits of 45 and 60 min. This is due to a portion of the population living too far from any BDC to be reached with the current network of hospitals under the parameters studied. We also observe that the drone's half range significantly affects reach. Only with the smallest time limit (30 min) do contrasts between the different half ranges become indistinguishable. This can be seen particularly in the overlapping curves in the subplot with a speed of 60 km/h, where all longer half ranges than 20 km overlap. This can be explained by the location of BDCs relative to trauma event locations, where short time spans do not allow for revealing differences in reach.

By comparing the proportion of reach in conjunction with the number of BDCs and half range simultaneously, we observe that increasing the half range can often compensate to varying extents for a smaller number of BDCs. This phenomenon is particularly evident with a time limit of 60 min and a speed of 100 km/h, where a reduction in the number of BDCs from 20 to 15 can be almost completely offset by increasing the half range from 50 to 60 km. It is noteworthy that the effect of drone speed is only apparent with combinations of small time limits and large half ranges. When the time limit is set to 60 min, the impact of speed is only marginal.

While the proportions of reach within certain time spans are clinically the most important measure, it is also worth considering the overall reach of the service, which is independent of drone speed and determined solely by the number of BDCs and the drone's half range. This is illustrated in Table 1. From this table, we observe that the reach increases systematically with both the number of BDCs and the half‐range values. Additionally, we observe that reducing the number of BDCs by five can be approximately compensated for by increasing the half range by 10 km, particularly at higher BDC values.

**TABLE 1:** Proportion of events within reach with different numbers of blood distribution centres (*N*) in use and various half‐range values of a drone.

Number of BDCs	*N* = 5	*N* = 10	*N* = 15	*N* = 20	*N* = 25
Half range = 20 km	0.21	0.28	0.36	0.39	0.44
Half range = 30 km	0.29	0.38	0.46	0.51	0.54
Half range = 40 km	0.34	0.46	0.56	0.62	0.64
Half range = 50 km	0.39	0.56	0.66	0.73	0.74
Half range = 60 km	0.45	0.65	0.74	0.81	0.83

Abbreviation: BDC, blood distribution centre.

From the simulations, we can also investigate the queue delays of deliveries in BDCs, which represent the additional time patients need to wait due to overlapping missions. This consideration is crucial when determining whether to deploy more than one operational drone per BDC. For simplicity, we have chosen here a specific set of drone parameters: speed = 100 km/h and half range = 50 km and the corresponding results are reported in Table 2 where we observe an overall decrease in total queue times, maximum queue time and average queue time as the number of BDCs increases beyond *N* = 10. This trend is expected due to the reduced spatio‐temporal overlap between missions. The observed increase in queue times from *N* = 5 to *N* = 10 can be attributed to the inclusion of longer‐distance missions, which initially causes a rise in queue times. However, this effect is offset by the growing number of available BDCs, leading to a subsequent reduction in overall queue times.

**TABLE 2:** Queue time characteristics with varying numbers of blood distribution centre (*N*) (speed = 100 km/h and maximum distance from blood distribution centre = 50 km).

Number of BDCs	*N* = 5	*N* = 10	*N* = 15	*N* = 20	*N* = 25
Yearly sum of queue times (min)	24.7	28.6	16.6	16.3	9.8
Max. queue time per mission (min)	22.0	23.7	15.9	15.5	9.8
Average queue time per mission (min)	0.024	0.028	0.017	0.016	0.010
Percent of queue times over 10/20/30 min	0.26 0.13 0.05	0.20 0.10 0.04	0.10 0.04 0.01	0.09 0.04 0.01	0.05 0.03 0.01

Abbreviation: BDC, blood distribution centre.

While the maximum queue times within a one‐year time interval are relatively large for a small number of BDCs, the average queue times remain consistently very small. Overall, the queue times are generally minimal, as indicated by the proportion of long waiting times (10/20/30 min).

In Table 3, we present the managerial implications of the implementation with varying numbers of BDCs. The overall airtime represents the average total airtime of drones across *N* BDCs over a one‐year period, including return flights. This quantity can be used, for example, to estimate the operational costs of drone flights. Mean total usage is computed as the fraction of overall airtime divided by minutes in *N* years (*N* * 365 * 24 * 60). The table also includes the total number of missions and mean mission lengths with varying values of *N*. From the table, we observe that the mean overall airtime initially increases with *N* and then decreases. This trend corresponds to the increased geographical coverage associated with higher *N*, as indicated by the rising mean number of missions. Conversely, a higher number of BDCs likely leads to shorter distances between hospitals and trauma scenes, which is seen in the shorter average mission lengths.

**TABLE 3:** Airtime characteristics with varying numbers of blood distribution centres (*N*) (speed = 100 km/h and maximum distance = 50 km).

Number of BDCs	*N* = 5	*N* = 10	*N* = 15	*N* = 20	*N* = 25
Overall airtime (in min)	9303	13,960	15,812	17,256	15,601
Total use (%)	0.354	0.265	0.201	0.164	0.118
Number of missions	376	534	633	697	708
Mean mission length (in min)	24.8	26.1	25.0	24.8	22.0

Abbreviation: BDC, blood distribution centre.

## DISCUSSION

In this study, we examined the implementation of whole blood delivery to trauma sites using drones in Finland. While parts of southern Finland are relatively densely populated, the overall population density is low. Our study illustrates how drones can be effectively utilized to deliver blood products in such a setting.

We found that, irrespective of the selected time limit, the number of BDCs and the drone's operational range are the most critical factors influencing patient reach. The drone's speed significantly impacts scenarios with the shortest time limits and has only a marginal effect with larger time limits. Additionally, it was observed that a smaller number of BDCs can largely be offset by increasing the drone's range. This finding is significant because enhancing a drone's range is much more cost‐effective than increasing the number of BDCs. Furthermore, advancements in battery technology make extending the operational range more feasible. With drones capable of half range of 60 km, 20 BDCs were typically able to cover over 80% of trauma events. This can seem slightly counterintuitive as, based on Figure 5 large geographical areas with trauma scenes are left uncovered with 25 BDCs, which at best corresponds to 74% coverage of trauma events. That is explained by the fact that a high proportion of the traumas happen near BDCs in highly populated areas, regardless of the general trend that the likelihood of traumas in proportion to population increases with rurality [18].

From the simulation, we observed that the overall occupancy rate of drones was less than 0.36%, and less than 0.75% for the most occupied BDC (see Table S3). This is consistent with a previous study dealing with occupancy rates of emergency helicopters in Finland [16]. The current simulations also assume only one drone per BDC. It is natural to consider whether BDCs should have more than one drone available for deliveries. Based on the simulation, queue times were very small, and therefore, additional drones would provide only marginal added value. Also, the overall usage of drones is generally very low, allowing for small maintenance tasks to be performed between missions, such as battery changes or loading the drone at the BDC, thereby ensuring high reliability in reach even with one drone. Overall, we do not see a need for additional drones in BDCs from the point of shortening the queue times in our idealized model. However, it is worth noting that with the current model, BDCs are somewhat differently occupied. For example, some BDCs serving highly populated areas of southern Finland could have a marginal benefit from an additional drone when the overall number of BDCs is low. This is further emphasized if potential realistic maintenance times between missions are long and cannot be performed without affecting availability for forthcoming missions.

Our simulation approach also assumed that drones fly back to the BDC immediately after delivery. An alternative implementation of drone deliveries is to utilize the drone's range fully for a one‐way delivery when additional range is needed. This can be achieved by changing the drone battery at the trauma scene for the return flight if needed. Another implementation is surface transport of a drone back to the sending BDC by car. However, both solutions require resources that can be difficult to allocate, and the magnitude of the delays is difficult to estimate. Therefore, we have excluded these considerations from our simulation model.

It should also be noted that the model does not account for weather conditions, which in practice can prevent drone deliveries or at least slow their speed and decrease range in difficult conditions. Additionally, some of the tested drone parameters, for example, speed 100 km/h and half range 50 km, are optimistic relative to currently used models. However, this is expected to change in the future, and there are already drones available on the market that meet these specifications [19].

While our model is a simplified version of reality, it provides insight into how drones could be effectively used for delivering blood products. Developing a simulation model that considers local weather conditions, the possible maintenance delays at the BDC and trauma site, the availability of medical staff to use the delivered blood products on site and other important practical factors is essential to fully understand the potential and requirements of using drones in prehospital blood transfers. Such a simulation model could be applied to any geographic area with available data on estimated delivery needs. Additionally, this type of simulation model could be used to address more dynamic situations involving spatio‐temporal changes in delivery needs, such as those caused by holiday seasons or catastrophic events.

## CONFLICT OF INTEREST STATEMENT

The authors declare no conflicts of interest.

## Supporting information


**Data S1.** Supporting information.

## Data Availability

The data that support the findings of this study are openly available in drone_sim at https://github.com/FRCBS/drone_sim.
