# Supplementary material for: Supplying whole blood with drones for prehospital transfusion at trauma sites in Finland: A simulation
Source: Vox Sang. 2025 Aug 13;120(10):1015–24. doi: 10.1111/vox.70092 (PMC12511842; doi:10.1111/vox.70092)
Supplement: Supplementary file 1 — Data S1. Supporting information. [file VOX-120-1015-s001.docx]

Supplementary material for: “Supplying whole blood with drones for prehospital transfusion at trauma sites in Finland - a simulation”

Panu Erästö^1^, Milla Juntunen^2^, Jukka Pappinen^3^, Jouni Nurmi^4^, Jarkko Ihalainen^2^, Jouni Lauronen^2^ & Mikko Arvas^2^

^1^Department of Information and Service Management, Aalto University School of Business, Espoo, Uusimaa, Finland

^2^ Finnish Red Cross Blood Service, Vantaa, Finland

^3^ Faculty of Medicine, Department of Public Health, University of Helsinki, Helsinki, Finland.

^4^ Emergency Medicine and Services, Helsinki University Hospital and University of Helsinki, Helsinki, Finland.

Corresponding author: Panu Erästö, panu.erasto@aalto.fi

[Supplementary Table 1 1](#_Toc882109891)

[Supplementary Table 2 2](#_Toc1547804750)

[Supplementary Table 3 4](#_Toc29592924)

[Supplementary Table 4 6](#_Toc606972380)

[Supplementary Table 5 8](#_Toc1148086918)

# Supplementary Table 1

| Supplementary Table 1: Selected BDCs with different total numbers of BDCs | | | | | |
| --- | --- | --- | --- | --- | --- |
| Hospital BDC | N=5 | N=10 | N=15 | N=20 | N=25 |
| Hyvinkää |  |  |  |  | x |
| Hämeenlinna |  |  | x | x | x |
| Joensuu |  |  | x | x | x |
| Jorvi | x | x |  |  | x |
| Jyväskylä |  | x | x | x | x |
| Kajaani |  |  |  | x | x |
| Kemi |  |  |  |  | x |
| Kokkola |  |  |  | x | x |
| Kotka |  | x | x | x | x |
| Kuopio |  | x | x | x | x |
| Lahti | x | x | x | x | x |
| Lappeenranta |  |  | x | x | x |
| Lohja |  |  | x | x | x |
| Meilahti |  |  |  |  | x |
| Mikkeli |  |  | x | x | x |
| Oulu | x | x | x | x | x |
| Peijas |  |  | x | x | x |
| Pori |  | x | x | x | x |
| Porvoo |  |  |  | x | x |
| Rovaniemi |  |  |  |  | x |
| Savonlinna |  |  |  | x | x |
| Seinäjoki |  | x | x | x | x |
| Tampere | x | x | x | x | x |
| Turku | x | x | x | x | x |
| Vaasa |  |  |  | x | x |

# Supplementary Table 2

| Supplementary Table 2: overall drone airtimes (mins) for each BDC with varying number of BDCs (N) (average speed=100 km/h and maximum one-way distance=50 km) with their corresponding occupancy rate | | | | | |
| --- | --- | --- | --- | --- | --- |
| Hospital BDC | N=5 | N=10 | N=15 | N=20 | N=25 |
| Hyvinkää |  |  |  |  | 731 |
| Hämeenlinna |  |  | 963 | 963 | 425 |
| Joensuu |  |  | 470 | 465 | 465 |
| Jorvi | 3928 | 3928 |  |  | 223 |
| Jyväskylä |  | 846 | 845 | 845 | 845 |
| Kajaani |  |  |  | 278 | 278 |
| Kemi |  |  |  |  | 243 |
| Kokkola |  |  |  | 439 | 439 |
| Kotka |  | 1019 | 1006 | 1006 | 908 |
| Kuopio |  | 706 | 706 | 706 | 706 |
| Lahti | 1472 | 1459 | 1044 | 1043 | 897 |
| Lappeenranta |  |  | 589 | 587 | 587 |
| Lohja |  |  | 1014 | 1014 | 813 |
| Meilahti |  |  |  |  | 432 |
| Mikkeli |  |  | 455 | 437 | 437 |
| Oulu | 635 | 635 | 635 | 635 | 634 |
| Peijas |  |  | 2972 | 2972 | 440 |
| Pori |  | 1193 | 1192 | 1192 | 1192 |
| Porvoo |  |  |  | 180 | 232 |
| Rovaniemi |  |  |  |  | 180 |
| Savonlinna |  |  |  | 374 | 374 |
| Seinäjoki |  | 906 | 905 | 757 | 757 |
| Tampere | 1757 | 1757 | 1522 | 1522 | 1522 |
| Turku | 1510 | 1510 | 1493 | 1493 | 1492 |
| Vaasa |  |  |  | 345 | 345 |
| Total sum | **9303** | **13960** | **15812** | **17256** | **15601** |

# Supplementary Table 3

| Supplementary Table 3: Total use (%) for each BDC with varying number of BDCs (N) (average speed=100 km/h and maximum one-way distance=50 km) | | | | | |
| --- | --- | --- | --- | --- | --- |
|  | N=5 | N=10 | N=15 | N=20 | N=25 |
| Hyvinkää |  |  |  |  | 0.08 |
| Hämeenlinna |  |  | 0.18 | 0.18 | 0.14 |
| Joensuu |  |  | 0.09 | 0.09 | 0.09 |
| Jorvi | 0.75 | 0.75 |  |  | 0.04 |
| Jyväskylä |  | 0.16 | 0.16 | 0.16 | 0.16 |
| Kajaani |  |  |  | 0.05 | 0.05 |
| Kemi |  |  |  |  | 0.05 |
| Kokkola |  |  |  | 0.08 | 0.08 |
| Kotka |  | 0.20 | 0.19 | 0.19 | 0.17 |
| Kuopio |  | 0.13 | 0.13 | 0.13 | 0.13 |
| Lahti | 0.28 | 0.28 | 0.20 | 0.20 | 0.17 |
| Lappeenranta |  |  | 0.11 | 0.11 | 0.11 |
| Lohja |  |  | 0.19 | 0.19 | 0.15 |
| Meilahti |  |  |  |  | 0.08 |
| Mikkeli |  |  | 0.09 | 0.08 | 0.08 |
| Oulu | 0.12 | 0.12 | 0.12 | 0.12 | 0.12 |
| Peijas |  |  | 0.57 | 0.57 | 0.08 |
| Pori |  | 0.23 | 0.23 | 0.23 | 0.23 |
| Porvoo |  |  |  | 0.03 | 0.04 |
| Rovaniemi |  |  |  |  | 0.03 |
| Savonlinna |  |  |  | 0.07 | 0.07 |
| Seinäjoki |  | 0.17 | 0.17 | 0.14 | 0.14 |
| Tampere | 0.34 | 0.34 | 0.29 | 0.29 | 0.29 |
| Turku | 0.29 | 0.29 | 0.28 | 0.28 | 0.28 |
| Vaasa |  |  |  | 0.07 | 0.07 |
| **Total sum** | **0.354** | **0.266** | **0.201** | **0.164** | **0.118** |

# Supplementary Table 4

| Supplementary Table 4: Mean number of missions for each BDC with varying number of BDCs (N) (speed=100 km/h and max distance=50 km) | | | | | |
| --- | --- | --- | --- | --- | --- |
| Hospital BDC | N=5 | N=10 | N=15 | N=20 | N=25 |
| Hyvinkää |  |  |  |  | 22 |
| Hämeenlinna |  |  | 31 | 31 | 25 |
| Joensuu |  |  | 20 | 20 | 20 |
| Jorvi | 162 | 162 |  |  | 21 |
| Jyväskylä |  | 34 | 34 | 34 | 34 |
| Kajaani |  |  |  | 12 | 12 |
| Kemi |  |  |  |  | 11 |
| Kokkola |  |  |  | 16 | 16 |
| Kotka |  | 31 | 31 | 30 | 28 |
| Kuopio |  | 29 | 29 | 29 | 29 |
| Lahti | 48 | 48 | 40 | 40 | 37 |
| Lappeenranta |  |  | 22 | 22 | 22 |
| Lohja |  |  | 31 | 31 | 25 |
| Meilahti |  |  |  |  | 70 |
| Mikkeli |  |  | 17 | 16 | 16 |
| Oulu | 37 | 37 | 37 | 37 | 37 |
| Peijas |  |  | 152 | 152 | 45 |
| Pori |  | 37 | 37 | 37 | 37 |
| Porvoo |  |  |  | 12 | 11 |
| Rovaniemi |  |  |  |  | 11 |
| Savonlinna |  |  |  | 12 | 12 |
| Seinäjoki |  | 28 | 28 | 25 | 25 |
| Tampere | 70 | 70 | 65 | 65 | 65 |
| Turku | 59 | 59 | 58 | 58 | 58 |
| Vaasa |  |  |  | 16 | 16 |
| **Total** | **376** | **534** | **633** | **697** | **708** |

# Supplementary Table 5

| Supplementary Table 5: Mean mission length (in min) for each BDC with varying number of BDCs (N) (speed=100 km/h and max distance=50 km) | | | | | |
| --- | --- | --- | --- | --- | --- |
| Hospital BDC | N=5 | N=10 | N=15 | N=20 | N=25 |
| Hyvinkää |  |  |  |  | 19.0 |
| Hämeenlinna |  |  | 31.2 | 31.2 | 29.0 |
| Joensuu |  |  | 23.1 | 22.9 | 22.9 |
| Jorvi | 24.3 | 24.3 |  |  | 10.6 |
| Jyväskylä |  | 25.1 | 25.1 | 25.1 | 25.1 |
| Kajaani |  |  |  | 23.2 | 23.2 |
| Kemi |  |  |  |  | 22.1 |
| Kokkola |  |  |  | 28.1 | 28.1 |
| Kotka |  | 33.2 | 33.0 | 33.0 | 31.8 |
| Kuopio |  | 24.5 | 24.5 | 24.5 | 24.5 |
| Lahti | 30.3 | 30.2 | 25.8 | 25.8 | 24.0 |
| Lappeenranta |  |  | 26.4 | 26.4 | 26.4 |
| Lohja |  |  | 32.8 | 32.8 | 32.2 |
| Meilahti |  |  |  |  | 6.2 |
| Mikkeli |  |  | 27.5 | 26.8 | 26.8 |
| Oulu | 17.2 | 17.2 | 17.2 | 17.2 | 17.2 |
| Peijas |  |  | 19.5 | 19.5 | 9.7 |
| Pori |  | 32.0 | 32.0 | 32.0 | 32.0 |
| Porvoo |  |  |  | 15.5 | 20.7 |
| Rovaniemi |  |  |  |  | 15.5 |
| Savonlinna |  |  |  | 31.0 | 31.0 |
| Seinäjoki |  | 32.1 | 32.0 | 29.8 | 29.8 |
| Tampere | 25.2 | 25.2 | 23.3 | 23.3 | 23.3 |
| Turku | 25.8 | 25.8 | 25.6 | 25.6 | 25.6 |
| Vaasa |  |  |  | 22.0 | 22.0 |
| **Overall** | **24.76** | **26.12** | **25.00** | **24.76** | **22.03** |
